# Kinase Function of Brassinosteroid Receptor Specified by Two Allosterically Regulated Subdomains

**DOI:** 10.3389/fpls.2021.802924

**Published:** 2022-01-13

**Authors:** Khawar Ali, Wenjuan Li, Yaopeng Qin, Shanshan Wang, Lijie Feng, Qiang Wei, Qunwei Bai, Bowen Zheng, Guishuang Li, Hongyan Ren, Guang Wu

**Affiliations:** College of Life Sciences, Shaanxi Normal University, Xi’an, China

**Keywords:** RLKs, brassinosteroids, BRI1, GSO1, dephosphorylation

## Abstract

Plants acquire the ability to adapt to the environment using transmembrane receptor-like kinases (RLKs) to sense the challenges from their surroundings and respond appropriately. RLKs perceive a variety of ligands through their variable extracellular domains (ECDs) that activate the highly conserved intracellular kinase domains (KDs) to control distinct biological functions through a well-developed downstream signaling cascade. A new study has emerged that brassinosteroid-insensitive 1 (BRI1) family and excess microsporocytes 1 (EMS1) but not GASSHO1 (GSO1) and other RLKs control distinct biological functions through the same signaling pathway, raising a question how the signaling pathway represented by BRI1 is specified. Here, we confirm that BRI1-KD is not functionally replaceable by GSO1-KD since the chimeric BRI1-GSO1 cannot rescue *bri1* mutants. We then identify two subdomains S1 and S2. BRI1 with its S1 and S2 substituted by that of GSO1 cannot rescue *bri1* mutants. Conversely, chimeric BRI1-GSO1 with its S1 and S2 substituted by that of BRI1 can rescue *bri1* mutants, suggesting that S1 and S2 are the sufficient requirements to specify the signaling function of BRI1. Consequently, all the other subdomains in the KD of BRI1 are functionally replaceable by that of GSO1 although the *in vitro* kinase activities vary after replacements, suggesting their functional robustness and mutational plasticity with diverse kinase activity. Interestingly, S1 contains αC-β4 loop as an allosteric hotspot and S2 includes kinase activation loop, proposedly regulating kinase activities. Further analysis reveals that this specific function requires β4 and β5 in addition to αC-β4 loop in S1. We, therefore, suggest that BRI1 specifies its kinase function through an allosteric regulation of these two subdomains to control its distinct biological functions, providing a new insight into the kinase evolution.

## Introduction

All living organisms sense and transduce signals through cell surface receptors to respond to the various challenges from the environment. Unlike animals, plants are more susceptible to these challenges due to their sessile nature. Through evolution over time, plants develop mechanisms that allow them to perceive different environmental signals *via* numerous sensory proteins and respond accordingly to survive and adapt. Receptor-like protein kinases (RLKs) are one of the most important and largest groups of transmembrane cell surface receptors in plants, which have more than 600 members in Arabidopsis alone, playing a fundamental role in intracellular and extracellular communications (Walker and Zhang, 1990; Walker, 1993; Shiu and Bleecker, 2001a). A typical RLK consists of three distinct functional domains: N-terminal extracellular domain (ECD) that binds a ligand, a transmembrane domain (TM) that anchors the protein within the membrane, and C-terminal intracellular kinase domain (KD) that transduces the signal downstream with serine–threonine–tyrosine specificity (Shiu and Bleecker, 2001a,b). During evolution, some of the RLKs have lost their ECD and TM, referred to as receptor-like cytoplasmic kinases (RLCKs) (Shiu and Bleecker, 2001a). Based on the phylogenetic analysis of their KDs and ECD structures, the RLKs are further divided into more than 40 subfamilies in *Arabidopsis thaliana*, of which the leucine-rich repeats receptor-like kinases (LRR-RLKs) are the largest one. LRR-RLK subfamily consists of 15 subgroups based on their KD similarities with more than 200 members in *Arabidopsis* (Shiu and Bleecker, 2001a; Liu et al., 2016, 2017). Based on their sequence similarities, expression profiles, biological functions, and interactions with other protein molecules, around 89 LRR-RLKs have been designated so far, and around 60 of them are functionally characterized (Wu et al., 2016).

Leucine-rich repeats receptor-like kinases control a wide range of biological functions in plants from growth and development to immunity and defense again pathogen and environmental stresses or sometimes both. For example, brassinosteroid (BR)-insensitive 1 (BRI1) is involved in BR signal transduction to activate the BR-response genes (Li and Chory, 1997; Wang et al., 2012). GASSHO1/2 (GSO1/GSO2) are required for the development of normal epidermal surface during embryogenesis and localization of Casparian strip proteins (Tsuwamoto et al., 2008; Pfister et al., 2014; Nakayama et al., 2017). Clavata1 (CLV1) and Barely ANY meristem1/2/3 (BAM1/BAM2/BAM3) control the apical meristem development (Clark et al., 1997; DeYoung et al., 2006) whereas HAESA (HAE) and HAESA-like2 (HSL2) regulate the floral organ abscission (Jinn et al., 2000; Cho et al., 2008). The excess microsporocytes 1 (EMS1) decide the anther development in Arabidopsis (Canales et al., 2002; Zhao et al., 2002) whereas the phytosulfokine receptor 1 (PSKR1) controls the hypocotyl length and cell expansion together with pathogen responses (Stührwohldt et al., 2011; Mosher et al., 2013). Similarly, a number of receptors are involved in defense against pathogens. For example, flagellin-sensitive 2 (FLS2) and EF-Tu receptor (EFR) contribute to innate immunity (Gomez-Gomez and Boller, 2000; Zipfel et al., 2006). BRI1-associated receptor kinase 1 (BAK1) is identified as a coreceptor that directly binds BRI1 in BR signaling (Li et al., 2002; Nam and Li, 2002). Later, it has been shown that a number of other receptors, such as FSL2, PSKR1, EFR, and peptide 1 receptor 1 and 2 (PEPR1 and PEPR2), also interact with BAK1 to provide the innate immunity to the plants (Chinchilla et al., 2007; Heese et al., 2007; Postel et al., 2010; Wang et al., 2015).

All the previous studies have shown that the LRR-RLKs need a ligand together with a coreceptor to initiate the downstream signaling pathways. This complex of ligand–receptor–coreceptor causes the conformational changes to start a signaling cascade of transphosphorylation downstream (Sun et al., 2013; Nolan et al., 2017). For instance, the BRI1 perceives plant growth hormone BRs at the cell surface, which causes the dissociation of BRI1 kinase inhibitor 1 (BKI1) that enables BRI1 and BAK1 to form a complex through transphosphorylation (Li and Nam, 2002; Wang et al., 2005, 2008). Following activation, the BRI1 phosphorylates its substrates, the BR signaling kinases (BSKs), and constitutive differential growth 1 (CDG1), which leads to the phosphorylation and activation of phosphatase *bri1* suppressor 1 (BSU1) (Mora-García et al., 2004; Kim et al., 2011). The activated BSU1 inhibits the GSK3/SHAGGY-like kinase brassinosteroid-insensitive 2 (BIN2) to release the specific transcription factor brassinozole-resistant 1 (BZR1) and bri1-EMS suppressor 1 (BES1) that regulate the expression of numerous BR-response genes (Li et al., 2001; He et al., 2002; Wang et al., 2002; Yan et al., 2009).

BRI1 belongs to the LRR-X subgroup of the LRR-RLKs family which regulates almost all the vital biological functions in the plants either directly or indirectly *via* cross-talks. Its function ranges from flowering time, male fertility, development of stomata, and root meristem development to the biotic and abiotic stress response and innate immunity (Jiang et al., 2013). The members of LRR-X subgroup, such as BRI1, BRI1-like 1 (BRL1), BRI1-like 2 (BRL2), and EMS1, share a high degree of similarity in their KDs. However, these receptors control distinct biological functions. Studies have shown that BRL1 and BRI1-like 3 (BRL3) can also directly bind the BRs, which leads to the activation of the same downstream signaling pathway (Cano-Delgado, 2004). A recent study reported for the first time that EMS1 shares the same downstream pathway with BRI1, offering a new insight into the molecular and functional evolution LRR-RLKs (Zheng et al., 2019). Distinct RLKs outside the LRR-X group, such as CLV1, HAE, and GSO1, are comparatively diverse in their KDs compared with LRR-X. GSO1 and GSO2 are the members of LRR-XI that control the normal development of embryo and localization of CASP protein (Tsuwamoto et al., 2008; Pfister et al., 2014). GSO1 and GSO2 are functionally redundant RLKs that perceive two peptide ligands, Casparian strip integrity factors 1 and 2 (CIF1 and CIF2) (Nakayama et al., 2017; Okuda et al., 2020). GSO1 and GSO2 double-mutant seedlings exhibit the adhesion between cotyledons, abnormal bending of embryo, and highly permeable epidermal structure (Tsuwamoto et al., 2008). Yet, how these receptors specify their intracellular function is unknown. With the intracellular signaling pathway well established, we planned to study how BRI1 diverges its function from other RLKs, that is, GSO1.

Both BRI1 and GSO1 belong to eukaryotic protein kinases (EPKs) shared a highly conservative structure, which functions as highly dynamic on-and-off switches rather than highly efficient catalysts to add a phosphate group to amino acid groups (Hanks et al., 1988; Manning et al., 2002; Taylor et al., 2019). This is largely attributed to the coinnovation of an activation fragment and allosteric regulations (Hanks et al., 1988; Kannan et al., 2007; Taylor et al., 2012, 2019). The activation segment can be activated by induced allostery through targeting substrates or tethering protein partners through allosteric hotspots (Remenyi et al., 2006; Deminoff et al., 2009; Gogl et al., 2019). Yet, how these regulations derive during the evolution to control specific intracellular signaling cascade remains elusive.

Using the domain-swap approach, here, we report that the chimeric receptor of BRI1 ECD fused GSO1-KD (BRI1-GSO1) cannot activate the intracellular signaling cascade shared with BRI1 since it was not able to rescue *bri1-301* mutant phenotypes. However, we have identified two significant functional domains in BRI1-KD such that they can replace the correspondent domains of BRI1-GSO1 to completely rescue the weak *bri1-301* and null *bri1-116* mutants back to the phenotypes, such as the wild type. Importantly, these chimeras activate the same downstream components as BRI1. Consistently, all the remaining motifs between BRI1 and GSO1 were functionally conserved and thus functionally interchangeable in terms of promoting plant growth, albeit varying in kinase activity *in vitro*. Since these two domains are known to be heavily regulated by allosteric regulation, our findings may provide a new insight into the kinase regulation and functional specification of BRI1, which might serve as a blueprint to study how other RLKs specify their intracellular functions to adapt to the new environmental challenges.

## Materials and Methods

### Plants Materials and Growth Conditions

The *Arabidopsis thaliana* ecotype Columbia (Col-0) was used as a wild type. The *bri1-301* and *bri-116* were used in Col-0 background. Seeds were surface-sterilized with 75% (v/v) ethanol for 10 min followed by a single wash of 2 min in 100% ethanol. The seeds were then washed with sterilized water two or three times and grown on 1/2 Murashige and Skoog (1/2 MS) medium with or without corresponding antibiotics. The seeds were stratified at 4°C for 2 days. Seven days after germination, the seedlings were transferred to the soil and grown under long-day conditions (16-h light/8-h dark) at 23°C.

### Vector Construction and Generation of Transgenic Lines

For the phenotypic complementation assays in *bri1-301* and *bri1-116* mutants, the full-length *BRI1* and *GSO1* receptors were amplified using primers that are listed in Supplementary Table 1 and fused with the binary vector *pCHF3:GFP* driven by BRI1 promoter. To construct the chimeric lines, the overlapping PCR strategy was implemented to combine the ECD of BRI1 with the KDs of *GSO1, BRL1, BRL2*, and *EMS1* to generate *BRI1-GSO1, BRI1-BRL1, BRI1-BRL2, and BRI1-EMS1* as reported previously (Zheng et al., 2019). The S1 and S2 motifs were exchanged between *BRI1* and *BRI1-GSO1* to generate the chimeras *GSO1^*BRI*1–S1^*, *GSO1^*BRI*1–S2^* and *BRI1^*GSO*1–S1^*, *BRI1^*GSO*1–S2^* using the same principle mentioned above. All the constructs were sequenced to verify. The resulting constructs were transferred into *Agrobacterium tumefaciens*, strain GV3101, through the freezethaw method (Weigel and Glazebrook, 2006) followed by plant transformation by the floral dip approach (Clough and Bent, 1998). Transgenic plants were screened on 1/2 MS medium supplemented with 45 ug/ml kanamycin. All the primers used in this study for the constructs are given in Supplementary Table 1.

### Hypocotyl and Root Growth Assay

To study the sensitivity of exogenous BR and PCZ in plant seedlings, the seeds were sterilized, washed, and germinated on 1/2MS media supplemented with 24-epibrassinolide (24-eBL, Sigma) at a concentration of 0 and 100 nM or with propiconazole (PCZ) at 0 and 5 μM. The seeds were allowed to grow vertically under light conditions for eBL and dark conditions for PCZ for 7 days. The seedlings were then photographed, and hypocotyl and root lengths were measured with the ImageJ program.

### Protein Extraction and Immunoblotting

Total protein extracts were extracted from the 14-day-old seedlings with 2 × SDS buffer (100 mM Tris, pH 6.8, 4% [w/v] SDS, 20% [v/v] glycerol, 0.2% [w/v] bromophenol blue, 2% [v/v] β-mercaptoethanol) by grinding in liquid nitrogen. The samples were centrifuged at 14,000 rpm for 10 min at 4°C. Supernatants were collected and separated on 10% SDS-PAGE gel and transferred into nitrocellulose (Pall Gelman) or PVDF membrane (Pall Gelman). The anti-GFP antibody (TRANSGEN) in 1:1000 dilution was used against GFP and the BES1 antibody (provided by Li Jia Lanzhou University, China) was used in 1:3000 dilution to quantify the phosphorylation status of BES1. HRP-linked goat anti-mouse antibody (Abcam) was used as secondary to quantify GFP whereas HRP-linked goat anti-rabbit antibody (Abcam) was used to detect the BES1 phosphorylation. Actin was used as a loading control (1:1000; Abmart).

### *In vitro* Kinase Assay

To determine *in vitro* kinase activity, the KDs of *BRI1, GSO1*, and all the chimeric receptors were cloned into a pGEXT-4T-3 vector to create GST-fused proteins. Overlapping PCR technique was employed to generate the *BRI1* and *GSO1* chimeric receptors. All the primers used for constructs that expressed proteins for *in vitro* kinase assay are listed in Supplementary Table 1. The constructs were transformed into *E. coli* strain BL21 (DE3), and the protein expression was induced by adding 0.5 mM isopropyl-β-D-thiogalactopyranoside (IPTG) into 300 ml liquid culture and then incubated overnight at 16°C. The expressed protein was purified with glutathione Sepharose 4B (GE Healthcare) according to the protocol provided by the manufacturer. For the phosphorylation assay, a 40 μl reaction buffer system (50 mM Tris–HCl, pH 7.4; 10 mM MgCl_2_; 150 mM NaCl and 1 mM ATP) was prepared for the equal amount of GST-fused protein and incubated at 37°C for 1 h. The protein samples were then boiled in 2 × SDS-PAGE loading buffer (62.5 mM Tris–HCl, pH 6.8, 2% [w/v] SDS, 10% [v/v] glycerol, 0.005% [w/v] bromophenol blue and 1% [v/v] β-mercaptoethanol) for 5 min at 95°C–100°C. Samples were separated on SDS-PAGE gel and transferred to a nitrocellulose membrane (Pall Gelman). The phosphorylation level was quantified by phosphothreonine antibody (anti-pThr; 1:2,000 dilution, CST #9381). The GST-tagged protein was detected by GST antibody (1:5,000 dilution, Proteintech # 66001-2-Ig).

### mRNA Expression Level by RT-PCR

Total mRNA was extracted from wild type, *bri1-301*, and transgenic plants using a plant RNA kit (OMEGA) according to the manufacturer’s protocol. The mRNA concentration was measured with a spectrophotometer. The cDNA was synthesized using a reverse transcriptase M-MLV First Strand cDNA Synthesis Kit (OMEGA) according to the manufacturer’s protocol. The cDNA was then diluted 10-fold with double-distilled water (ddH_2_O). The Phanta^®^ Super-Fidelity DNA Polymerase (Vazyme) was used to perform PCR amplification for the *DWF4, CPD, BAS1*, and *ACT* from the cDNA templates. The primers used for the RT-PCR are given in Supplementary Table 1.

### *bri1-116* Background Genotyping

Total genomic DNA was extracted from the leaves of 30-day-old plants of Col-0, *bri-116*, and transgenic lines in the *bri1-116* background using the DNA extraction buffer (500 mM Tris–HCl pH 8, 100 mM EDTA, 5 mM NaCl, and 10% SDS) by grinding in liquid nitrogen. After 10 min of incubation at 80°C, the mixture of chloroform/isoamyl alcohol/ethanol was added in a 20:1:4 ratio. The mixture was then centrifuged at 12,000 rpm and the supernatant was extracted. The DNA was precipitated by adding chilled isopropyl alcohol and centrifuged. The DNA pallet was washed two times in 75% ethanol by 12,000 rpm. The mutant *bri1-116* gene was amplified from the genomic DNA using Super-Fidelity DNA Polymerase (Vazyme) by the specific genotyping primers given in Supplementary Table 1. The amplified DNA was digested with *Pme*I (NEB) restriction enzyme and incubated for four h. *Pme*I restriction enzyme produced double cuts on Col-0 and single cut on *bri1-116* mutant producing three bands for Col-0 with 179 kb, 368 kb, and 1509 kb and two bands for *bri1-116* with 547 kb and 1509 kb.

### Alignment Construction and Phylogenetic Analysis

*Arabidopsis thaliana* LRR-RLK sequences were retrieved from “the Arabidopsis Information Resource,”^1^ as described previously (Shiu et al., 2004). Multiple protein sequence alignments for the KDs were generated using MAFFT^2^ (Katoh and Standley, 2013). The phylogenetic tree for BRI1 and its homologs and other *Arabidopsis* LRR-RLK were made with IQ-TREE^3^ (Trifinopoulos et al., 2016). For each tree, an appropriate model was automatically selected using Bayesian information criterion (BIC) in IQ-Tree. SH-aLRT strategy with approximate Bayes test and 1000 replicates were conducted to obtain branch support values for each internal node of the tree. All the trees were visualized using FigTree v 1.4.4 program.^4^ For the crystal structure analysis, the X-ray structures of BRI1 (5LPZ) was downloaded from protein database bank^5^ (Berman et al., 2000) and GSO1 (C0LGQ5) from AlphaFold protein structure database^6^ and then visualized and labeled using PyMOL Molecular Graphic System.^7^

### Statistical Analysis

Statistical analysis was performed using one-way of variance (ANOVA), two-way analysis of variance (ANOVA), and Tukey’s test, as implemented in GraphPad Prism 9.0 (GraphPad Software^8^).

## Results

### The GASSHO1 and Chimeric Brassinosteroid-Insensitive 1-GASSHO1 Have no Brassinosteroid Function

**FIGURE 1:**
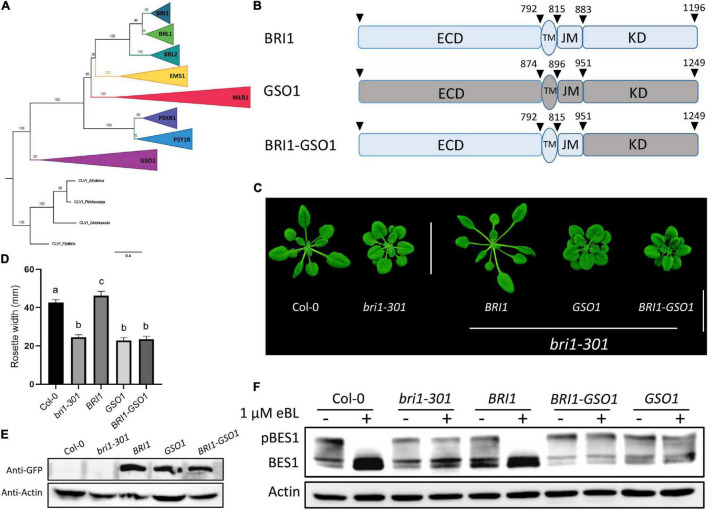
GSO1 and BRI1 ECD fused with GSO1 KD (BRI1-GSO1) could not complement *bri1* mutant. **(A)** The phylogenetic relationship between the BRI1 group members with GSO1 was estimated using maximum likelihood with 1000 bootstrap replicates with CLAVATA1 used as an outgroup. The values on the branches represent the bootstrap values. **(B)** Schematic representation of BRI1, GSO1, and GSO1 KD fused with BRI1 ECD. The numbers above the receptor represent the starting position of each domain in the receptors. **(C)** Phenotypes of BRI1, GSO1, and chimeric BRI1-GSO1 receptor in *bri1-301* mutants. Scale bar = 2.0 cm. **(D)** Comparison of rosette width of 4-week-old plants (*n* = 15), *p* < 0.0001, one-way ANOVA with a Tukey’s test. Different letters indicate significant differences. **(E)** Protein expression level of transgenic lines from panel **(C)**. **(F)** BES1 dephosphorylation status. No dephosphorylation of BES1 was observed in GSO1 and chimeric BRI1-GSO1 receptor indicative of no BR signaling. Three independent experiments were performed with similar results.

The previous studies have already demonstrated that the non-BR receptor EMS1 activates the same transcription factor BES1 and BZR1 downstream, suggesting that different receptors that bind different ligands can still use the same machinery to control different biological functions (Chen et al., 2019; Zheng et al., 2019). Yet, PSKR1 and GSO1 that encode 1008 and 1249 amino acids, respectively, cannot activate BR signaling. GSO1 includes 31 LRRs in its ECD, along with its TM and KD. The phylogenetic analysis based on the conserved KDs puts GSO1 into subgroup LRR-XI, which is one of the biggest subgroups of LRR-RLKs out-grouped with LRR-X (Tsuwamoto et al., 2008; Pfister et al., 2014; Figure 1A and Supplementary Figures 1, 2). Since PSKR1 was in LRR-X and GSO1 was in LRR-XI, for better separation, we selected GSO1 for studying how BRI1 specifies its function from GSO1. We found that the full-length GSO1 under *BRI1* promoter (*pBR:GSO1*) could not complement the mutant phenotype of *bri1-301* and the rosette width of the transgenic plants was similar to that of *bri1* mutants (Figures 1C–E). We confirmed that the *pBR:BRI1-GSO1* (BRI1-GSO1) were not able to rescue to the dwarf phenotype of *bri-301* (Zheng et al., 2019). Conversely, we showed that *pBR:BRI1-BRL1* (*BRI1-BRL1*), *pBR:BRI1-BRL2* (*BRI1-BRL2*), and *pBR:BRI1-EMS1* (*BRI1-EMS1*) completely rescued the *bri1* mutant phenotypes, confirming the functional divergence in BRI1 and GSO1 (Figures 1B–E and Supplementary Figures 3A–C). To assure this finding, we examined the phosphorylation of BES1 since dephosphorylation BES1 is a very specific hallmark of activation of the BR signaling pathway (Yin et al., 2002). We thus examined the phosphorylated vs. dephosphorylated ratios of BES1 in the transgenic lines. Upon BR treatment, we detected no accumulation of dephosphorylated BES1 in GSO1 and chimeric BRI-GSO1 transgenic lines when compared to the wild type or BRI1 that accumulated dephosphorylated BES1 (Figure 1F).

### C Motifs Are Functionally Conserved Between Brassinosteroid-Insensitive 1 and GASSHO1

**FIGURE 2:**
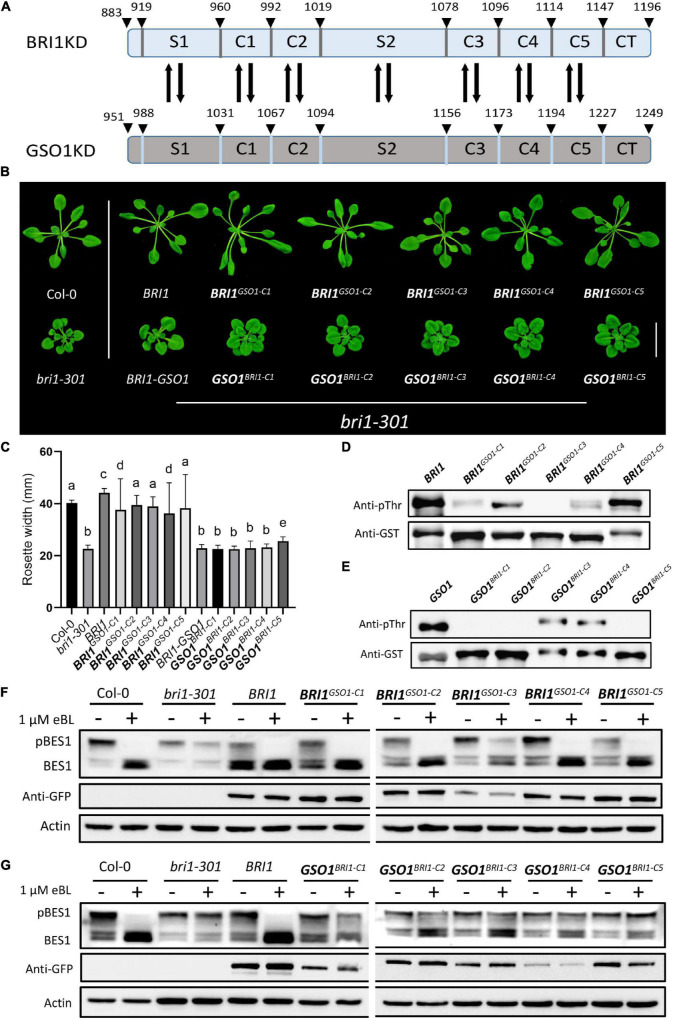
The C motifs are functionally conserved in biology but diverse in kinase activity. **(A)** Schematics of BRI1 and GSO1 KDs motifs showing the positions for domain swap. The number above represents the positions of amino acids residues on KDs. **(B)** Phenotypic analysis of wild type, *bri1-301*, and transgenic lines expressing chimeric receptors. Biologically functional conservation for the C domain of *BRI1* during evolution. Scale bar = 2.0 cm. **(C)** Rosette width comparison of lines shown in panel **(B)** (*n* = 15), *p* < 0.0001, one-way ANOVA with Tukey’s test. Different letters indicate significant differences. **(D,E)** Phosphorylation activity of GST-fused with the KDs of BRI1 and *BRI1^*GSO*1–C^* chimeras, GSO1 and *GSO1^*BRI*1–C^* chimeras. The anti-pThr antibody was used to detect the autophosphorylation levels. GST-tagged proteins detected by anti-GST antibody served as a loading control. All the experiments were repeated independently three times with similar results. **(F,G)** Immunoblotting of eBL induced dephosphorylation of wild type, *bri1-301*, BRI1 and transgenic lines of *BRI1^*GSO*1–C1^* *BRI1^*GSO*1–C2^* *BRI1^*GSO*1–C3^* *BRI1^*GSO*1–C4^* *BRI1^*GSO*1–C5^* and *GSO1^*BRI*1–C1^* *GSO1^*BRI*1–C2^* *GSO1^*BRI*1–C3^* *GSO1^*BRI*1–C4^* *GSO1^*BRI*1–C5^*. An anti-GFP antibody was used to quantify the protein expression. Actin served as a loading control.

All the RLKs descended from a common ancestor and expanded through gene duplication and divergence (Shiu and Bleecker, 2001b). Following duplication, most commonly, the duplicated genes are eliminated from the genome but in some cases, mutations can be accumulated and fixed over time, which leads to the functional divergence between the duplicated genes (Moore and Purugganan, 2005; Innan and Kondrashov, 2010). GSO1, PSKR1, EMS1, and BRL2 originate in land plants whereas BRL1 derives in seed plants and BRI1 derives in flowering plants. Therefore, BRI1 is a derived RLK and GSO1 is an ancestral RLK (Supplementary Figure 1). To find some clues for their functional divergence, we constructed multiple sequence alignments in plants between the BRI1 family and the GSO1 members to identify the conserved residues because sequence divergence is a good indicator of functional divergence. From our multiple sequence alignments, we discovered that the identified conserved residues in the BRI1 family were diverged with the GSO1 members and localized to two key positions, which we later identified as αC-β4 loop and activation loop (Supplementary Figures 5, 6). Based on the divergent residues, we divided the KD into S and C motifs. The “C” represented the sequence-conserved motifs that have less divergent residues, whereas “S” represented the sequence-diverse domains that have most divergent residues between the BRI1 family and GSO1 (Figure 2A and Supplementary Figures 5, 6). Then, we applied the domain-swap strategy to construct chimeras of these motifs in BRI1 and BRI-GSO1. We used BRI1 as a control to see whether these motif substitutions could alter the BRI1’s function.

First, we substituted the C motifs between BRI1 (*BRI1^*GSO*1–C1^*, *BRI1^*GSO*1–C2^*, *BRI1^*GSO*1–C3^*, *BRI1^*GSO*1–C4^*, *BRI1^*GSO*1–C5^*) and BRI1-GSO1 (*GSO1^*BRI*1–C1^*, *GSO1^*BRI*1–C2^*, *GSO1^*BRI*1–C3^*, *GSO1^*BRI*1–C4^*, *GSO1^*BRI*1–C5^*) and expressed all the chimeras in *bri1-301* mutants under *BRI1* promoter (Figure 2A and Supplementary Figure 6). We showed that the substitution of C motifs from GSO1 into BRI1 (*BRI1^*GSO*1–C^*) did not drastically alter the BRI1 function and all the chimeras could function normally since they all completely restored the *bri1* mutant phenotypes. As expected, none of the chimeras of BRI1-GSO1 (*GSO1^*BRI*1–C^*) had gained the ability to rescue the mutant phenotypes when expressed in *bri1-301*. One possible explanation is that the function of C motifs is conserved, consistent with their sequence conservation (Figures 2B,C). Autophosphorylation is a widespread mechanism regulating kinase functions (Beenstock et al., 2016). In addition, BRI1 autophosphorylation and transphosphorylation act as a molecular switch and play an essential role in BR-regulated plant growth and development (Xu et al., 2008; Oh et al., 2009). We thus examined the *in vitro* autophosphorylation of BRI1, GSO1, and all the chimera as shown in Figure 2B. We found that except BRI1^GSO1–C3^, all other chimeras, BRI1^GSO1–C1^, BRI1^GSO1–C2^, BRI1^GSO1–C4^, and BRI1^GSO1–C5^ more or less showed kinase activity (Figure 2D). On the other hand, GSO1^BRI1–C1^, GSO1^BRI1–C2^, and GSO1^BRI1–C5^ showed no kinase activity, but GSO1^BRI1–C3^ and GSO1^BRI1–C4^ showed a mild kinase activity, suggesting that the kinase activities were somewhat asymmetrically altered after swapping any C domains, although the cause remains to be addressed (Figure 2E). Interestingly, the BES1 dephosphorylation assay showed that all the *BRI1^*GSO*1–C^* chimeras accumulated dephosphorylated BES1 but not the *GSO1^*BRI*1–C^* chimeras, which is consistent with our phenotypic results (Figures 2F,G). Taken together, BRI1-KD possesses mutational plasticity and functional robustness, namely different genotypes with similar biological phenotypes (functions).

**FIGURE 3:**
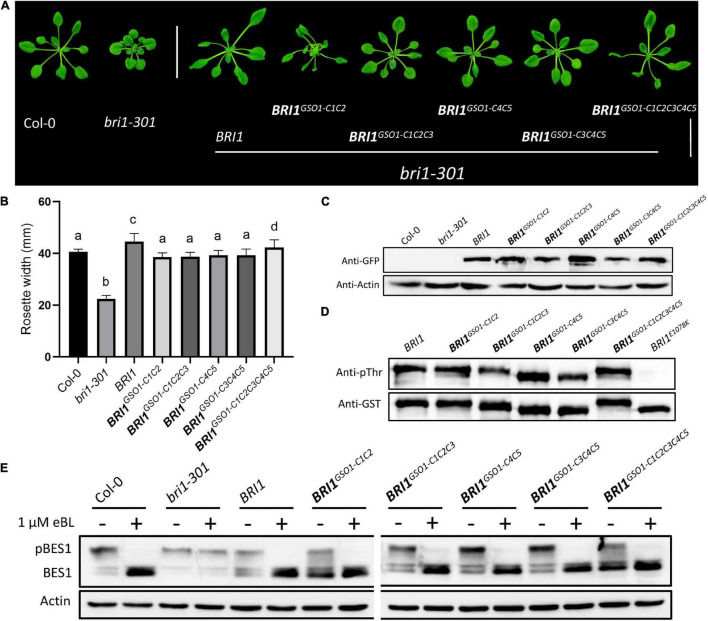
Functional conservation in the C domains of BRI1. **(A)** phenotypes of 4-week-old transgenic lines representing the expression of functionally conserved chimeric receptors. Scale bar = 2.0cm. **(B)** Measurement of rosette width of transgenic lines (*n* = 15), *p* < 0.0001, one-way ANOVA with a Tukey’s test. **(C)** Transgenic lines depicting protein expression profile. Anti-GFP was used to quantify the protein and actin served as a loading control. **(D)** *In vitro* kinase assay of GST-fused BRI1 and its chimeric proteins using an anti-pThr antibody. Anti-GST antibodies served as a loading control. Kinase-null mutant BRI1^E1078K^ was used as a negative control, and all the experiments were repeated independently three times with similar results. **(E)** BES1 dephosphorylation assay of BRI1 and its transgenic lines treated with or without eBL used in panel **(A)**. Actin used as a loading control.

We further speculated that a conserved single motif might not be sufficient to enhance or knock out the BR signaling. We thus designed different combinations of C motif and expressed them in *bri1* mutants to enhance the effects on the phenotypes. Interestingly, we had a similar finding as to the single motif replacements (Figures 2, 3). All the different combinations of *BRI1^*GSO*1–C^* functioned normally by recovering the *bri1* mutant to the wild type. Importantly, all the C motifs together generated *BRI1^*GSO*1–C1*C*2*C*3*C*4*C*5^* that showed similar results (Figures 3A–E). Altogether, these results suggest that during evolution, the C motifs remained functionally conserved between BRI1 and GSO1, consistent with our speculation that BRI1 possesses mutational plasticity and functional robustness.

### Motif S1 and S2 Specify the Functional Divergence Between Brassinosteroid-Insensitive 1 and GASSHO1

**FIGURE 4:**
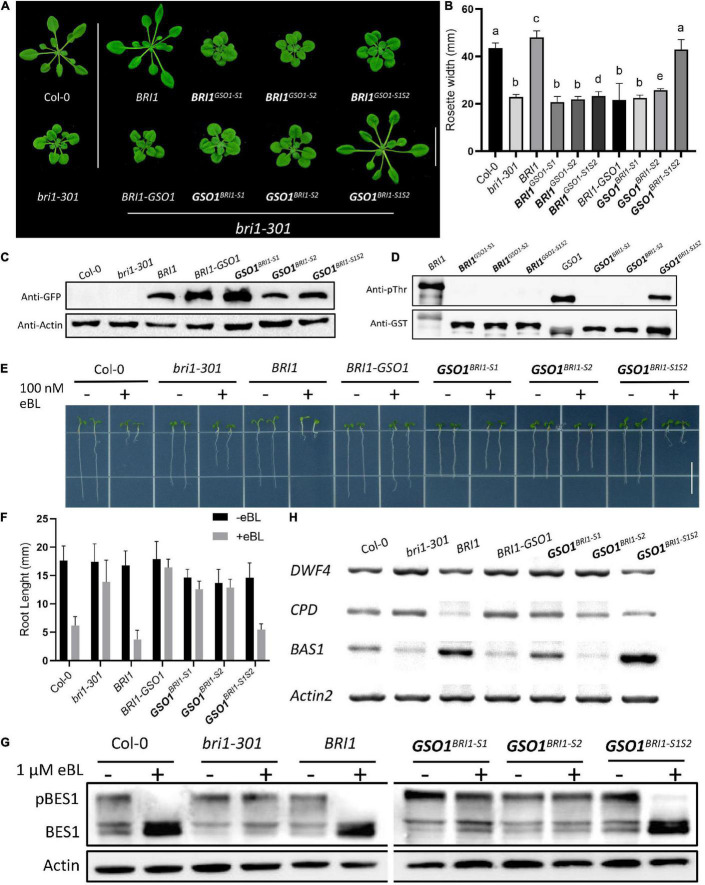
The kinase function of BRI1 defined by two separate domains. **(A)** The phenotypes of transgenic lines expressing different chimeric receptors of BRI1 and GSO1. Only chimeric receptors with both the S1 and S2 domains of BRI1 (*BRI1* and *GSO1^*BRI*1–^*^S1*S*2^) rescued the mutant phenotypes but not the others (*GSO1^*BRI*1–S1^*, *BRI1^*GSO*1–S1^*, *BRI1^*GSO*1–S2^*, and *GSO1^*BRI*1–S2^*). Scale bar = 2.0 cm. **(B)** Measurement of rosette width of transgenic lines (*n* = 15), *p* < 0.0001, one-way ANOVA with a Tukey’s test. **(C)** The protein expression level of corresponding lines. Actin served as a loading control. **(D)** Phosphorylation of GST-fused BRI1, GSO1, and their chimeras. The anti-pThr antibody was used to detect the autophosphorylation level. GST-tagged proteins were detected by anti-GST antibody served as a loading control. **(E)** The root of the 7-day-old seedling grown under light conditions on 1/2MS media supplemented with eBL (24-epibrassinolide). Scale bar = 1.0 cm. **(F)** Comparison of root length in wild type, *bri1-301*, and transgenic lines (*n* = 13). **(G)** The dephosphorylation levels of BES1. The total protein was extracted from 14-day-old seedlings, and phosphorylated (pBES1) and dephosphorylated BES1 were detected with BES1 antibodies. Actin served as a loading control. **(H)** Expression levels of BR biosynthetic genes, *DWF4, CPD*, and *BAS1* in wild type, *bri1-301*, and transgenic lines. *ACT* served as a loading control.

We argue that if the C motifs are functionally conserved between BRI1 and BRI1-GSO1, then the S motifs must affect functional divergence between BRI1 and BRI1-GSO1. To test this hypothesis, we substituted the BRI1 S1 and S2 with GSO1 S1 and S2 to make the *BRI1^*GSO*1–S1^* (919–960) and *BRI1^*GSO*1–S2^* (1019–1078) chimera and expressed under the *BRI1* promoter to rescue the *bri1* mutant phenotypes (Figure 2A). Surprisingly, we found that BRI1 function was completely lost in transgenic lines and was not able to rescue *bri1* mutant phenotypes (Figures 4A,B). Next, we substituted the BRI1-GSO1 S1 and S2 with BRI1 S1 and S2 to make the *GSO1^*BRI*1–S1^* (988–1031) and *GSO1^*BRI*1–S2^* (1094–1156) chimeras and expressed them in *bri1* mutants. We found that neither *GSO1^*BRI*1–S1^* nor *GSO1^*BRI*1–S2^* complemented the *bri1* mutant phenotypes (Figures 4A–C). Through these findings, we speculate that both S1 and S2 are required for BRI1 to function, and by replacing either of these will lead to the complete loss of BRI1 function but are not sufficient for the chimeric BRI1-GSO1 receptor to activate BR-dependent signaling. To address this problem, we substituted both S1 and S2 motifs together into BRI1-GSO1 to make *GSO1^*BRI*1–S1*S*2^*. To our surprise, it completely restored the weak *bri1-301* and a null *bri1-116* mutant phenotypes to the wild type by activating the BR signaling (Figures 4A–C and Supplementary Figures 4A–C). Consistently, the BRI1 *in vitro* autophosphorylation assay showed that only the GSO1^BRI1–S1S2^ was sufficient to specify the kinase activity of BRI1 but not the BRI1^GSO1–S1^, BRI1^GSO1–S2^, BRI1^GSO1–S1S2^, GSO1^BRI1–S1^, and GSO1^BRI1–S2^ (Figure 4D).

To test whether the *GSO1^*BRI*1–S1*S*2^* indeed is BR-dependent, the seeds were grown on 1/2 MS media with or without brassinosteroid, 24-eBL or PCZ, a BR biosynthetic inhibitor (Asami et al., 2000) to various concentrations and found that the transgenic lines expressing *GSO1^*BRI*1–S1*S*2^* had similar sensitivity to the exogenous BR as that of the expression of *BRI1* in *bri1* mutants or wild-type plants (Figures 4E,F and Supplementary Figures 4D,E). Similarly, the transgenic lines expressing *GSO1^*BRI*1–S1*S*2^* also showed sensitivity to PCZ and the hypocotyls were inhibited as the same as wild type (Supplementary Figures 4F,G). Conversely, the transgenic lines of *BRI1-GSO1*, *GSO1^*BRI*1–S1^*, and *GSO1^*BRI*1–S2^* showed no altered sensitivity to the BL or PCZ (Figures 4E,F and Supplementary Figures 4D–G). We also examined the accumulation status of dephosphorylated BES1, and as expected, high levels of dephosphorylated BES1 were detected in *GSO1^*BRI*1–S1*S*2^* transgenic lines, whereas no accumulation of BES1 dephosphorylation activity was observed in individual *GSO1^*BRI*1–S1^* or *GSO1^*BRI*1–S2^* (Figure 4G). These results indicate that the *GSO1^*BRI*1–S1*S*2^* is BR-dependent and can act as a functional receptor for the BR. Taken together, we have identified that motif S1 and motif S2 together are sufficient requirements to specify the kinase function of BRI1 whereas each of them is a necessary but not a sufficient requirement.

To further explore our findings, we examined the expression level of BR biosynthetic genes, such as *CPD* and *DWF4*, and a BR catabolic gene, *BAS1*. In activated BR signaling, the BR biosynthetic genes are downregulated whereas the BR catabolic genes are upregulated since they are subjected to feedback regulations (Wang et al., 2002; Yin et al., 2002; Clouse, 2011). We found that the *CPD* and *DWF4* were drastically downregulated whereas the *BAS1* was upregulated in *GSO1^*BRI*1–S1*S*2^* transgenic lines (Figure 4H). These findings indicate that the chimeric *GSO1^*BRI*1–S1*S*2^* can trigger similar BR responses as *BRI1*. These results also confirm that BRI1 requires both S1 and S2 to function since replacing either of them can lead to the complete knockout of the BR function. Taken together, our results suggest that the S1 and S2 motifs define the function of BRI1.

**FIGURE 5:**
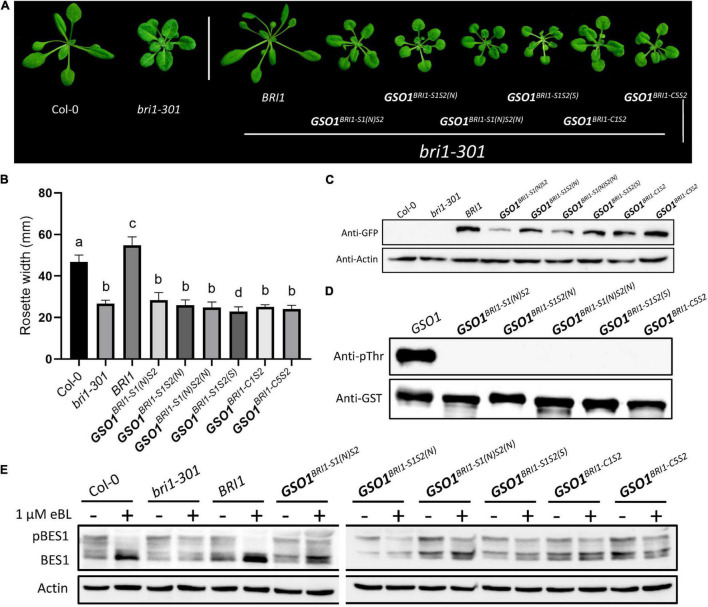
Defining the necessary regions of S1 and S2. **(A)** Phenotypic expression of different chimeric *BRI1-GSO1* transgenic lines that are different from the original motif-S1 and motif S2 in their sequence position on KD. The (N) represents the new sequence position whereas (S) represents the short segment on KD. Their specific sequence limits were marked in Supplementary Figure 6. Scale bar = 2.0 cm. **(B)** Quantification of 4-week-old plants (*n* = 13), *p* < 0.0001, one-way ANOVA with Tukey’s test. **(C)** The protein expression level of corresponding lines is shown. Actin served as a loading control. **(D)** *In vitro* kinase activity assay of GST-fused recombinant chimeric protein of GSO1 KD. **(E)** Immunoblotting of eBL induced dephosphorylation of BES1 in wild type, *bri1-301*, BRI1, and chimeric lines in the *bri1-301* background.

To approximate the key structural requirements for the function of BRI1-KD in S1 and S2, we shrank or changed the position for either of the motifs to form the new chimeras of *GSO1^*BRI*1–S1(N)S2^*, *GSO1^*BRI*1–S1*S*2(N)^*, *GSO1^*BRI*1–S1(N)S2(N)^*, and *GSO1^*BRI*1–S1*S*2(S)^* and expressed them in *bri1-301* mutants (Supplementary Figure 6). Interestingly, we found that neither motifs were shrinkable to complement the mutant phenotype of *bri1*. In addition, no accumulation of BES1 dephosphorylated activity or kinase activity was observed. Furthermore, we also combined two C motifs together with S motif (*GSO1^*BRI*1–C1*S*2^* and *GSO1^*BRI*1–C5*S*2^*) to test the specificity of S1 and S2 and got similar results as that of S1 and S2 alone, respectively, suggesting that β4 and β5 in motif S1 and activation loop in motif S2 are required for BRI1-KD to allosterically specify the BR-dependent signaling and kinase activity (Figures 5A–E).

### Motif S1 and S2 Constitute Crucial Regulatory Domains

**FIGURE 6:**
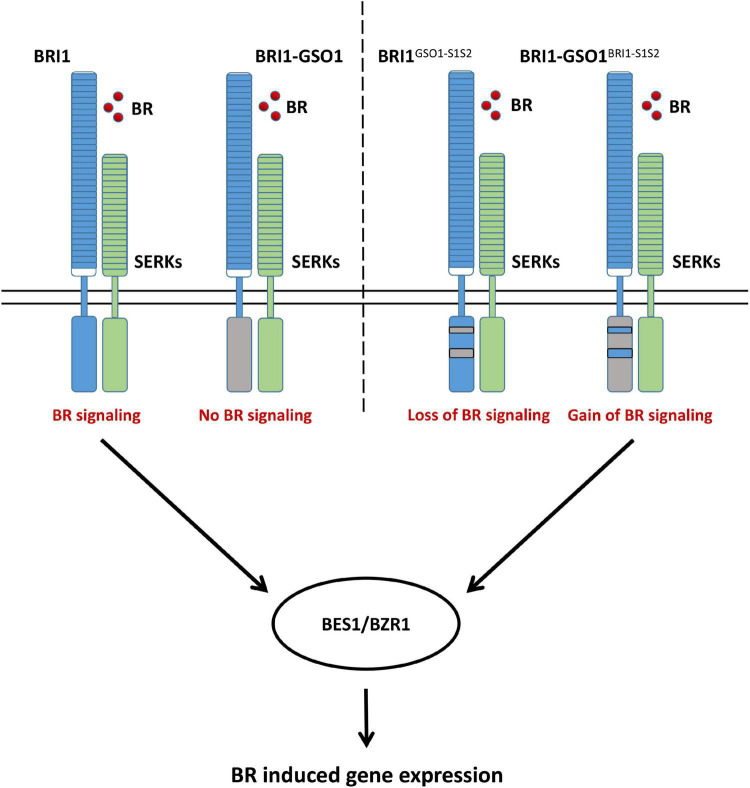
A proposed model illustrating how BRI1 specifies its function from GSO1, which leads to the control of distinct biological function and how a domain swap from BRI1 into chimeric BRI1-GSO1 can completely restore the BR signaling pathway.

The secondary and crystal structures of EPKs have shown that the regulatory kinase core of EPKs consists of an N-lobe, an activation loop, and a C-lobe (Bojar et al., 2014; Zhang et al., 2015). Our secondary structure comparison of BRI1 and GSO1 revealed that one of the regulatory hotspots αC happens to be located at the very beginning of the motif S1 of the N-lobe (Supplementary Figure 6). αC domain is responsible for the conformational changes of the kinase core by forming a Glu-Lys salt bridge and a domain closer between the N- and C-lobes (Beenstock et al., 2016). However, since S1(N) includes αC but without β4 and β5 cannot perform the same function as S1, β4 and β5 play a role to specify the function of BRI1-KD (Figure 5). Furthermore, S2(N) without the activation loop in the motif S2 cannot perform the same function as S2 (Figure 5). Thus, the activation loop plays a critical role in the activation of kinases by autophosphorylation, making it the most important region of the main kinase core of the EPKs (Supplementary Figure 6). In most EPKs, the lack of activation loop autophosphorylation makes the catalytically impotent kinases (Beenstock et al., 2016). In conclusion, our results suggest that the S1 together with S2 are the necessary requirements to specify the function of BRI1 from GSO1 (Figure 6). Furthermore, β4, β5, and activation loop are critical for the allosteric regulation of S1 and S2.

## Discussion

Receptor-like kinases are one the most important groups of plant cell surface receptors that arise from a common ancestor, playing a critical role in plant growth and development. Their unique structure makes them suitable for cell-to-cell communication. Since the discovery of the first RLK in maize, functional studies of RLKs have become ever-growing research field in plant biology (Walker and Zhang, 1990). There are more than 600 and 1131 RLK members in *Arabidopsis* and rice, respectively (Shiu and Bleecker, 2001a,b; Shiu et al., 2004). Plants evolve these many RLKs to possibly meet the everyday challenges that they face from the environments and invasion of pathogens. The RLKs allow plants to sense and cope with these challenges to survive. One of the largest subgroups of RLKs is LRR-RLK that has repetitive leucine residues in their ECD to sense a ligand on the cell surface. So far, only a small number of LRR-RLKs have been well characterized. Among them, the BRI1 family has been studied extensively.

The ECD of RLKs is highly diverse, which allows them to perceive various kinds of ligands to control different biological functions. The KD, on the other hand, is conserved among all RLKs. The BRI1 and EMS1 belong to the same LRR-RLK-X group, acquiring this ability to control diverse functions *via* perceiving different ligands but targeting the same downstream components (Zheng et al., 2019). We applied the same strategy to the receptor GSO1, a distant out-group of BRI1 family (Figure 1A and Supplementary Figures 1, 2). Using the domain-swap strategy, the ECD of BRI1 was fused with the KD of GSO1 to make a chimeric receptor BRI1-GSO1 (Figure 1B). The functional complementation of *bri1* mutants with *GSO1* shows that neither *GSO1* nor chimeric *BRI1-GSO1* can restore the *bri1* mutant phenotypes, indicating that GSO1-KD has a function distinct from that of BRI1-KD (Figures 1C–E). Furthermore, both *GSO1* and *BRI1-GSO1* failed to promote the accumulation of dephosphorylated BES1 in their transgenic lines, consistent with the phenotypic results (Figure 1F). Together, these results support the notion that BRI1 and GSO1 are a result of RLK duplication, divergence, and expansion *in planta* to adapt to the present need through their KDs (Ohta, 1989; Innan and Kondrashov, 2010).

During evolution, new genes evolve through duplication and then functional differentiation. If the newly evolved genes are beneficial to the survival of the plants, they will be retained through selection otherwise deleted from the genomes (Ohno, 1970; Hughes, 1994; Force et al., 1999). The EPKs are one of the largest protein families with a common ancestor in eukaryotes, yet all EPKs have a similar structure, representing one of the most conserved protein families (Hanks et al., 1988; Manning et al., 2002). This raises a question, how the EPKs rapidly duplicate and diversify with such highly conserved sequences and structures.

The EPKs are considered dynamic molecular switches that control numerous biological functions through transferring a Ɣ-phosphate from ATP to the free OH group of serine/threonine or tyrosine residue of the substrate protein (Taylor et al., 2012; Zhang et al., 2015; Beenstock et al., 2016). The RLKs are orthologous to Drosophila Pelle kinase and human interleukin-1 receptor-associated kinases (IRAKs) that resemble the animals tyrosine kinases (RTKs) in their domain organization (Cock et al., 2002; Lehti-Shiu et al., 2012). The protein kinase structural organizations and their role in conformational regulation have been studied extensively in eukaryotes. Two flexible elements that undergo dramatic conformational changes upon activation are proposed to include αC-helix and activation loop (Zhang et al., 2006; Taylor et al., 2012; Bojar et al., 2014). Our comparison of secondary and crystal structures of BRI1 and GSO1 shows that S1 and S2 contain these important regulatory domains, a regulatory hotspot that includes αC-β4 loop and activation loop in N-lobe and C-lobe, respectively. We show that both S1 and S2 are required for the autophosphorylation and activation of the kinase function of BRI1 *in planta* (Supplementary Figure 7). Interestingly, in spite of having important regulatory domains, neither motif is sufficient to induce the activation of the kinase. But together, they are able to activate the autophosphorylation, possibly implying an allosteric regulation between these two motifs, a common mechanism of distal regulation of macromolecules (Zhang et al., 2006, 2015; Hu et al., 2013).

Genetic studies clearly suggest that S2, the activation loop in particular, is important for the function of BRI1 and probably for other EPKs or RLKs as well since most intracellular genetic mutants of BRI1 are mapped to the activation loop (Sun et al., 2017; Supplementary Figure 6). We uncover that an activation loop is required for the functional specificity of S2 since *GSO1^*BRI*1–S1*S*2^* that includes the activation loop of BRI1 can rescue *bri1* mutants but *GSO1^*BRI*1–S1*S*2(N)^* that has the activation loop of GSO1 instead cannot (Figure 4 and Supplementary Figure 4). This means that the activation loop of BRI1 is crucial to specify its function from GSO1. Compared the activation loops of BRI1 and GSO1, we observe at least nine replacements in the conserved residues in the BRI1 family drastically different from that of GSO1, that is, A1036e, H1040n, L1041t, S1042d, V1043s, S1044n, L1046w, G1048c, and P1050y (small letters for correspondent residues in GSO1) (Supplementary Figure 6). Interestingly, there are two genetic mutants of BRI1, G1048D (*bri1-115*), and P1050S (*bri1-702*), identified in these residues, implying a possible role of these divergent residues in specifying the function of BRI1 from other RLKs (Supplementary Figure 6). Similarly, *GSO1^*BRI*1–S1*S*2^* that includes the β4 and β5 of BRI1 can rescue *bri1* mutants but *GSO1^*BRI*1–S1(N)S2^* that has the β4 and β5 of GSO1 instead cannot, implying a crucial role of β4 and β5 to specify BRI1 from GSO1. By comparison of β4 and β5 between BRI1 and GSO1, we uncover at least four replacements in the conserved residues of BRI1 family drastically different from that of GSO1, that is, P941k, K947s, G949k, and R952n (small letters for correspondent residues in GSO1) (Supplementary Figure 6). Surprisingly, there is only one genetic mutant identified in the whole S1. It is even more surprised that this genetic mutant is in these divergent residues rather than in the residues conserved across the selected RLKs. It is not less significant that R952W (*bri1-202*) is a strong *bri1* mutant. Among all the missense genetic mutants identified in eleven sites of the KD of BRI1, only five mutants locate in residues not absolutely conserved across the selected RLKs (Sun et al., 2017; Supplementary Figure 6). One of them locates in β4 and β5 of S1 and two of them locate in the activation loop of BRI1 of S2 (Supplementary Figure 6). This does not appear as a random distribution, consistent with the idea that the β4 and β5 of S1 and activation loop of S2 play a significant role in the function and evolution of BRI1-KD. Although the αC-β4 loop between the αC and β4 loop has been identified as an allosteric hotspot, which can work together with the activation loop to regulate kinase activities and malfunctions in αC-β4 loop cause diseases in human, its role in RLKs is unknown. Our study should be a step stone to investigate its functional and evolutionary roles in RLKs and EPKs. Furthermore, the role of β5 has not been reported in EPKs and our data reveal its significance in the function and evolution of BRI1-KD. The future study should be able to address its broad significance in BRI1 and other RLKs and EPKs.

In eukaryotes, protein phosphorylation is one of the initial steps which is crucial for the coordination of cellular and organic functions, such as growth and regulation metabolism, subcellular trafficking, proliferation, apoptosis, inflammation, and many other physiological processes. In human alone, there are over 500 protein kinases maintaining the cellular function (Taylor et al., 2012). Due to their crucial role in many biological processes, EPKs play a central role in many diseases’ progression, such as cancer and other epigenetic-related diseases. To date, more than 1,000 variations in kinase protein expression have been reported in various human tumors (Ardito et al., 2017). Most of these tumor-related alterations belong to the kinases from the closest animal counterpart RTKs, such as RON, FGFR1-4, IGF-1R, ALK, c-Ret, c-Met, and HER-2 (Bhullar et al., 2018). Since kinase specificity plays a major role in cancer development, protein kinase-targeted drugs have emerged as the most efficient way of cancer treatment, and currently more than 70 drugs based on tyrosine inhibition are used for several cancer treatments (Cohen et al., 2021). However, many of these kinase inhibitors are associated with off-target effects and toxicities, such as proteinuria, hypothyroidism, cardiotoxicity, and skin reactions (Orphanos et al., 2009; Shah et al., 2013). This is because that almost all the kinase inhibitors used for medical treatments are targeting ATP-binding sites that are conserved across all EPKs. On the other hand, kinase inhibitors recognize and target specific domains on the protein kinases, such as αC-β4 loop that connects the αC helix to the β8 strand and reported to have a diverse combination of cancer-related mutation (Supplementary Figure 6; Yeung et al., 2020). Therefore, kinase inhibitors act through the αC-β4 loop or β5 can be highly specific. Hence, our study can illuminate a study in this area and beyond.

The robustness—the capacity to preserve the existent functions—and the evolvability—the ability to acquire novel functions—are two essential properties in all biological systems to counter the enrichment of deleterious mutations (Bershtein et al., 2006). At first glance, robustness and evolvability operate in the opposite directions of evolution such that when natural selection is dominant, it purges deleterious mutations (Lenski et al., 2006). However, deleterious mutations can be restrictive mutations that potentially become advantageous by subsequent mutations through epistasis, providing a stepping-stone for adaptations. Thus, natural selection increases the robustness but decreases the evolvability (Stiffler et al., 2015; Zheng et al., 2020). Conversely, when genetic drift is dominant, it retains abundant deleterious mutations. As such, genetic drift decreases the robustness but increases the evolvability (Zheng et al., 2020). However, robustness permits the accumulation of neutral mutations that can be fixed by subsequent mutations if they become beneficial, generating many-to-one redundancy in genotype-phenotype maps, which increases evolvability. Therefore, robustness and evolvability become a paradox needed to resolve in biological systems (Lenski et al., 2006; Wagner, 2008; Mayer and Hansen, 2017; Zheng et al., 2020). Whereas our major effort is to identify how evolution specifies the function of BRI1-KD, we have not neglected the fact that the KDs of RLKs are highly conserved. We notice that besides the two S domains, the exchange of C motifs does not significantly alter the biological functions of BRI1 since *BRI1^*GSO*1–C^* chimeras alone or together completely restore *bri1* phenotypes to the wild type with high-level accumulation of dephosphorylated BES1 when treated with BRs, although the *in vitro* kinase activities vary (Figure 2). This means that the KD has high functional robustness in spite of genetic changes, which then can promote the evolution of S1 and S2, thus likely promoting the evolvability. Therefore, our study implies that the KDs of RLKs can reconcile robustness and the evolvability, yet this is an area remained to be investigated in the future.

Previously, we have shown that the exchange of KDs between BRI1 and EMS1 allows them to perform their functions in their respective niches (Zheng et al., 2019), meaning that a different ligand can trigger the same downstream signaling. We now show that we can change the function of GSO1-KD by mere two small domains (S1 and S2) with that of BRI1-KD to acquire a function similar to that of BRI1-KD. This means that the function of a native RLK can be easily changed, suggesting that the same ligand can trigger different downstream signaling after a minimal domain swap. This could potentially impact future crop biotechnology such that it allows more abundant or less expensive ligands to activate a pathway used to control by less abundant or more expensive ligands. For example, BRs are relatively expensive compared with sugars. In plants, there are a large class of extracellular motifs containing lectins found in RLKs. These lectin receptor protein kinases (LecRKs) can bind various sugars (Shiu and Bleecker, 2001a). If we can convert their KDs to function as BRI1-KD, then we may construct a BR signaling pathway in crops without needing BRs. Therefore, it is a great potential of saving in crop production without fertilization, which seems not totally out of reach if our findings can sustain future scrutiny. Hence, this is a promising area for future exploration.

## Data Availability Statement

Sequence data for this article can be downloaded from the TAIR, Phytozome v12 listed in this section under the following accession numbers: BRI1 AT4G39400, GSO1 AT4G20140, BRL1 AT1G55610, BRL2 AT2G01950, EMS1 AT5G07280, ACT2 AT3G18780, CPD AT5G05690, DWF4 AT3G50660, and BAS1 AT2G26710.

## Author Contributions

GW and KA conceived, designed, and coordinated the project. KA, WL, YQ, SW, and LF performed molecular cloning and plasmid construction. KA performed protein expression and purification. KA, WL, and QW performed *in vitro* kinase assay and western blots and quantified gel bands. KA and WL performed the bioinformatics analysis. GW, KA, WL, QW, QB, GL, BZ, and HR interpreted the results. GW and KA wrote the original draft and other authors read and edited the manuscript.

## Conflict of Interest

The authors declare that the research was conducted in the absence of any commercial or financial relationships that could be construed as a potential conflict of interest.

## Publisher’s Note

All claims expressed in this article are solely those of the authors and do not necessarily represent those of their affiliated organizations, or those of the publisher, the editors and the reviewers. Any product that may be evaluated in this article, or claim that may be made by its manufacturer, is not guaranteed or endorsed by the publisher.

## Footnotes

1
https://www.arabidopsis.org/

2
https://www.ebi.ac.uk/Tools/msa/mafft/

3
http://iqtree.cibiv.univie.ac.at/

4
http://tree.bio.ed.ac.uk/software/figtree/

5
https://www.rcsb.org/

6
https://www.alphafold.ebi.ac.uk/

7
https://pymol.org

8
http://www.graphpad.com
