# Supplementary material for: Kinase Function of Brassinosteroid Receptor Specified by Two Allosterically Regulated Subdomains
Source: Front Plant Sci. 2022 Jan 13;12:802924. doi: 10.3389/fpls.2021.802924 (PMC8792736; doi:10.3389/fpls.2021.802924)
Supplement: Supplementary file 1 [file Data_Sheet_1.pdf]

Supplementary Figures and Tables

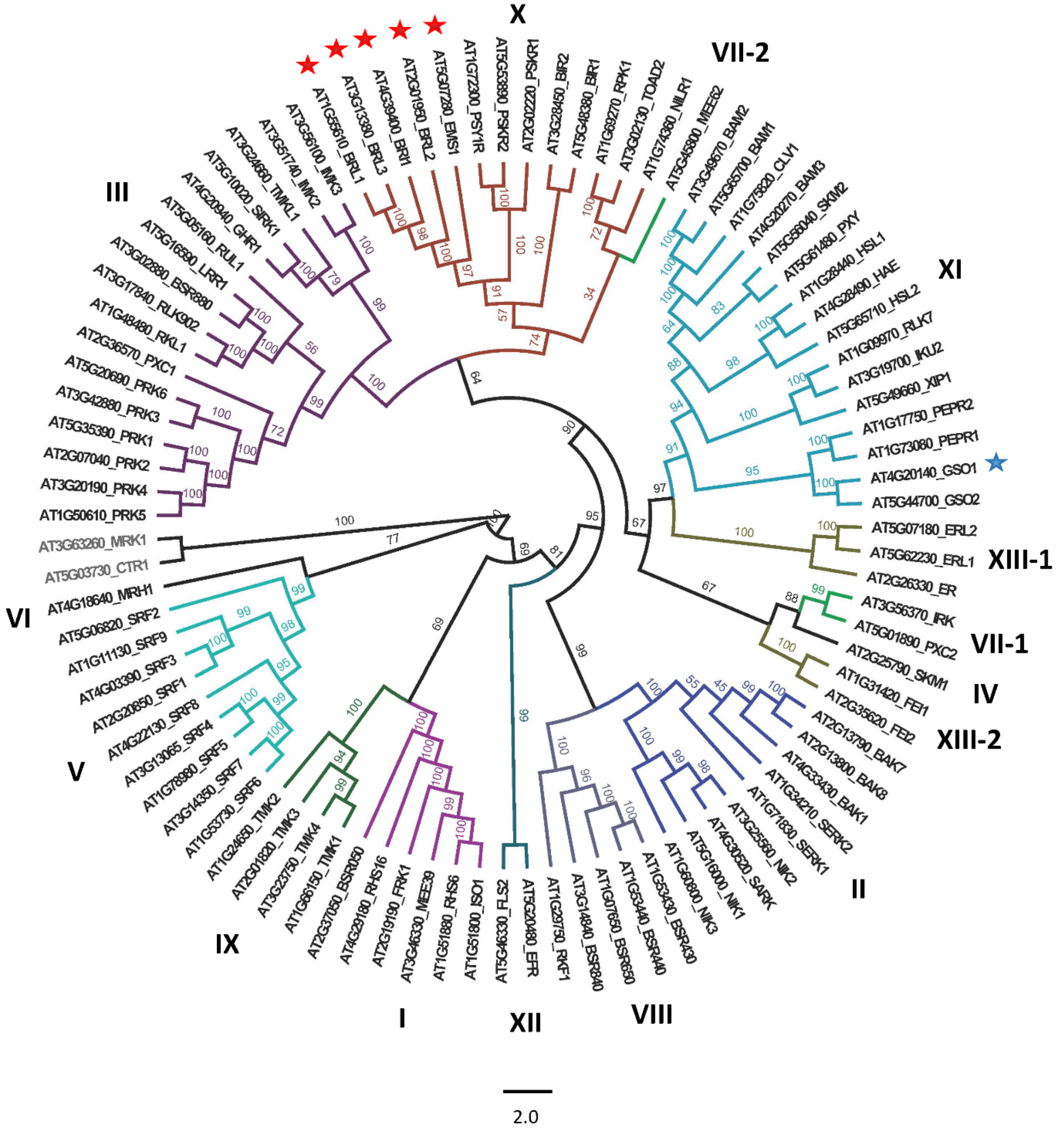

Supplementary Figure 1| Phylogenetic analysis of designated *Arabidopsis thaliana* LLR-RLKs. The kinase domain sequences were aligned with MAFFT, and the tree was inferred by the maximum likelihood (ML) method with 1000 bootstrap using IQ-TREE software. The roman character represents the group number while the number on the branches represents the bootstrap values. The red stars denote the

BRI1 family, and the blue star represents the GSO1. CTR1 and MRK1 were used as an outgroup.

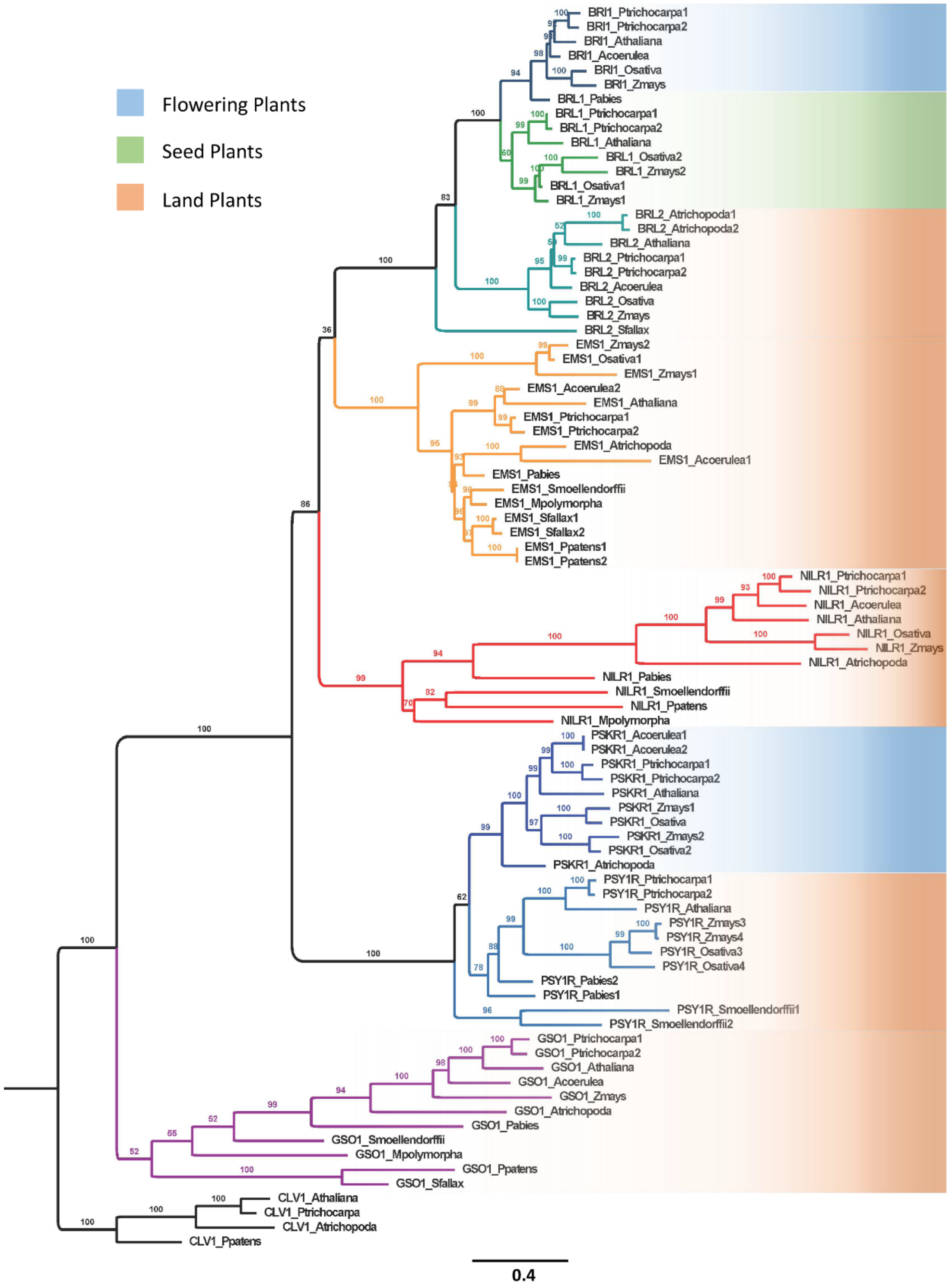

Supplementary Figure 2| The expanded phylogenetic tree of BRI1 group members with GSO1 as shown in **(Figure 1A)**. The sequences were aligned with MAFFT, and the tree was inferred by the maximum likelihood (ML) method with 1000 bootstrap using IQ-TREE software. The branch colors represent different gene families, while

the group colors represent their occurrence in plant species. The number on the branches represents the bootstrap values. CLAVATA1 was used as an outgroup.

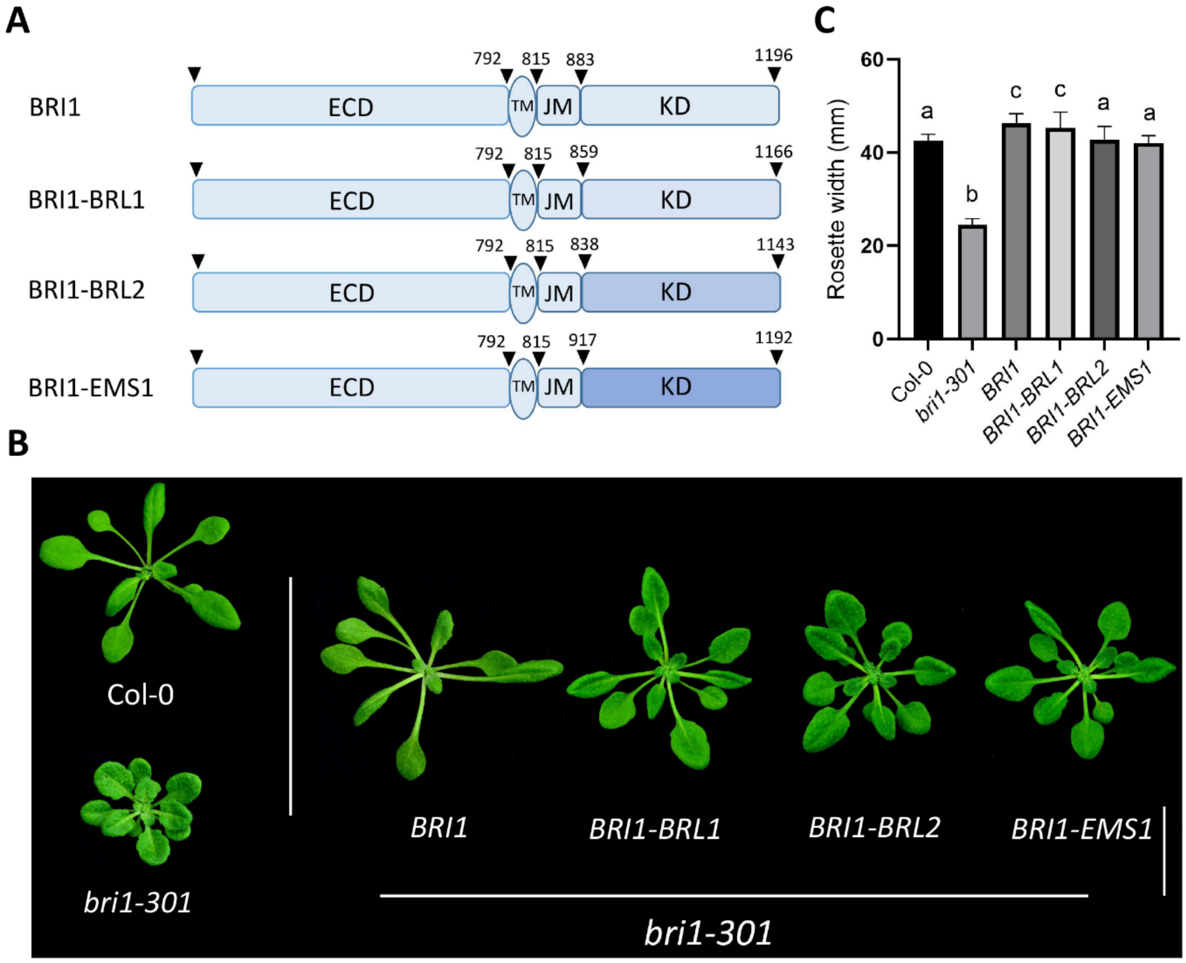

Supplementary Figure 3| BRI1 ECD fused with KDs of BRL1 (*BRI1-BRL1*), BRL2 (*BRI1-BRL2*), and EMS1 (*BRI1-EMS1*) completely restored the mutant phenotype to the wild type. **(A)** Schematic representation of *BRI1, BRI1-BRL1*, *BRI1-BRL2*, and *BRI1-EMS1* chimeras. The numbers above schematic diagrams represent the positions of each domain within the receptors. **(B)** Phenotypes of *BRI1, BRI1-BRL1*, *BRI1-BRL2*, and *BRI1-EMS1* chimeric receptors in *bri-301* background. Scale bar = 2.0 cm. **(C)** Comparison of rosette width of 4-weeks-old plants (n=15), *P*<0.0001, one-way ANOVA with a Tukey's test. Different letters indicate significant differences.

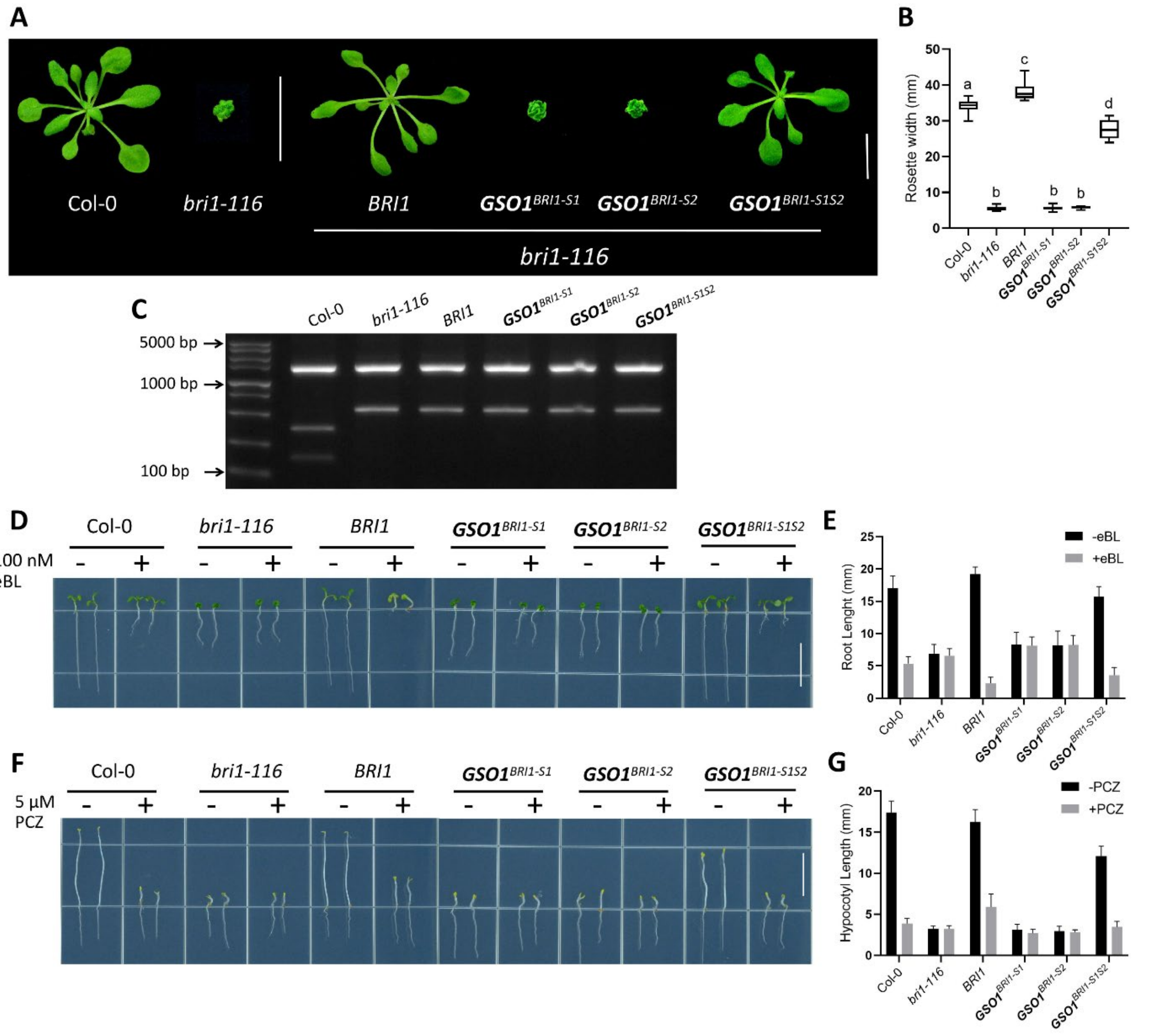

Supplementary Figure 4| The $GSO1^{BRI1\text{-S1S2}}$ chimera rescues *bri1* null mutant *bri1-116* phenotypes. **(A)** Phenotypes of 4-week-old transgenic lines expressing chimeric receptors under *BRI1* promoter in *bri1-116* background. Scale bar = 1.0 cm. **(B)** Comparisons of rosette width of plants as shown in (**A**) (n=13), P<0.0001, one-way ANOVA with a Tukey's test. **(C)** Genotyping of Col-0, *bri1-116***,** and transgenic lines as shown in **(A)**. **(D, F)** Comparisons of plants treated with or without eBL (24-epibrassinolide) for root length **(D)** or PCZ (BR biosynthesis Inhibitor) for hypocotyl length measurements **(F)**. The seedlings were grown vertically on 1/2 MS medium with or without 100 nMeBL for 7 d in light conditions or with or without 5 µM PCZ for

5 d in darkness. **(E, G)** Comparisons of root length (n=15) **(E)**, and hypocotyl elongation (n=15) **(G)** in *bri1-116* background.

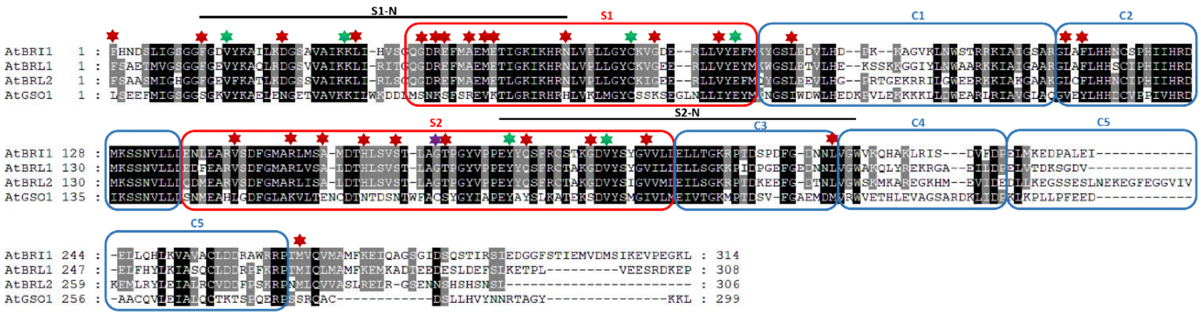

Supplementary Figure 5| Distribution of residues conserved for RLKs with BR signaling but diverged from GSO1. The red stars represent the residues conserved in BRI1 family but diverged from GSO1. The green stars represent the residues conserved across BRI1 family and GSO1, while the purple star indicates the *bri1-115* (G1048D) mutant in the above-mentioned conserved residue of BRI1.

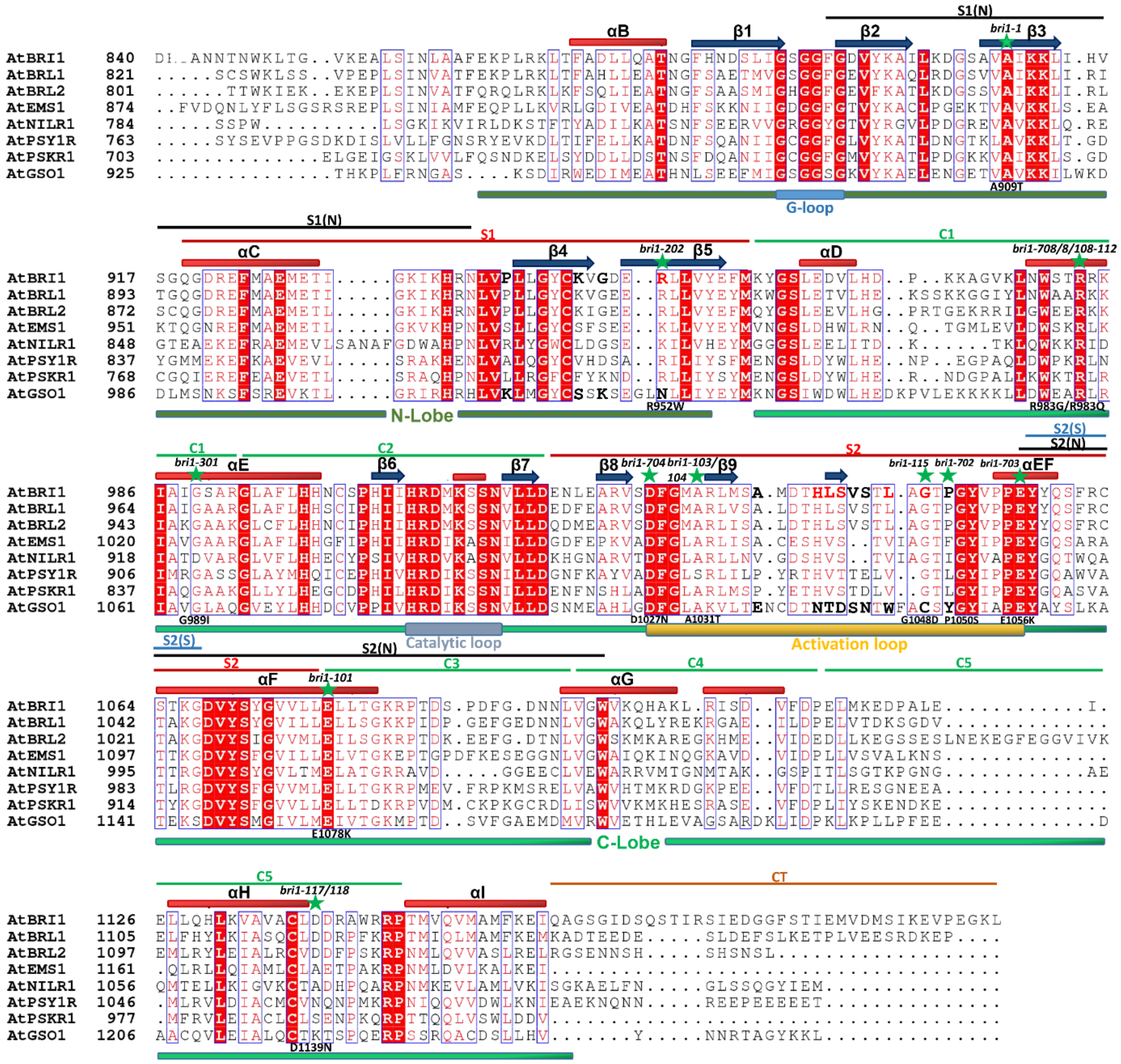

Supplementary Figure 6| Protein sequence alignment of BRI1 group and GSO1 from *Arabidopsis thaliana* (At). The functional elements are labeled above and below the sequences. The red lines above the sequences represent the functionally diversified S1 and S2 domains. The green lines denote the functionally conserved C domains. The black lines indicate the new S motifs while the blue represents the truncated S2. The blue arrows represent the β-strands, the red columns represent α-helixes and the green stars are missense mutation sites.

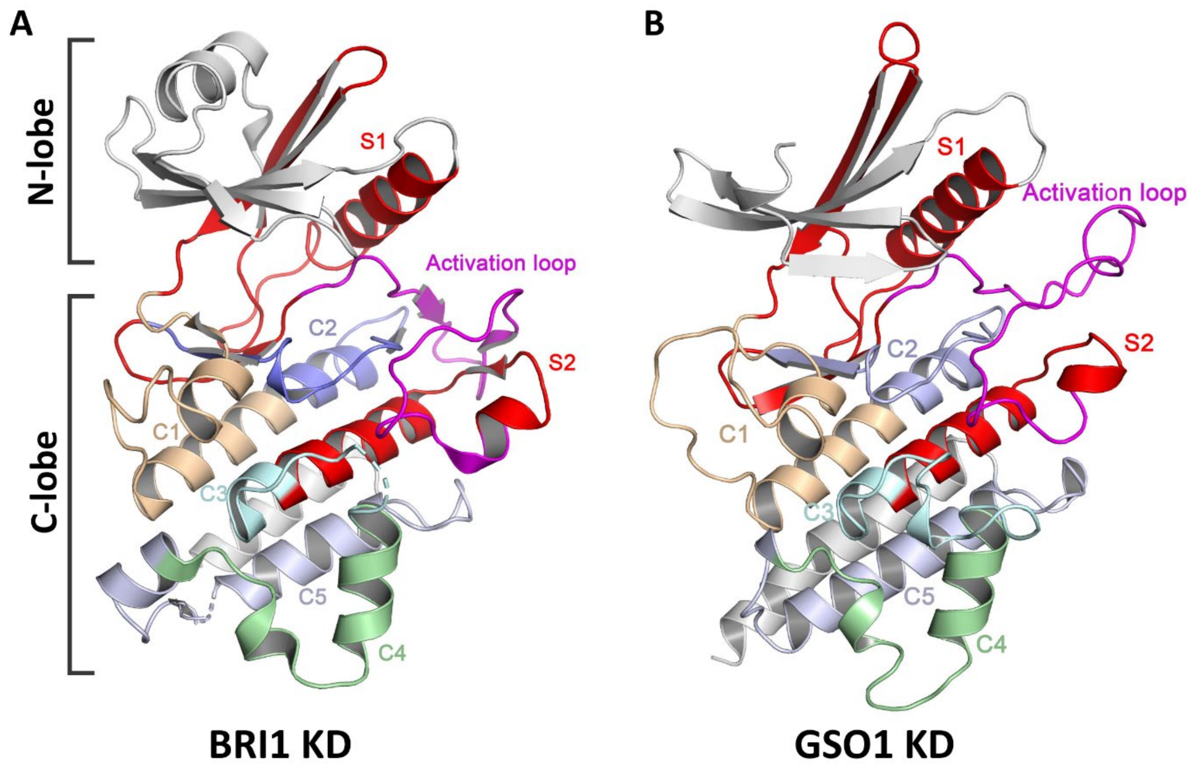

Supplementary Figure 7| Conserved structural domains of BRI1 and GSO1. **(A, B)** Crystal structures of BRI1 (5LPZ) **(A)** and GSO1 (C0LGQ5) **(B)**. The S1 and S2 are depicted in red color while the activation loop is presented in purple color. C motifs are depicted in various colors in N-lobe and C-lobe.

Supplementary Table 1| Primers used in this study

Gene cloning and overlapping	
BRI1-F-KpnI	GGTACCATGAAGACTTTTTCAAGCTTCTTTCTCT
BRI1-R-BamHI	GGATCCTAATTTTCCTTCAGGAACTTCTTTTATACT
GSO1-F-KpnI	GGTACCATGCAACCACTAGTTCTTCTCCTCCT
GSO1-R-BamHI	GGATCCCAGCTTCTTATAACCGGCCGTTCTGT
BRI1-GSO1-F1	GGCTACCAATGGTCTAAGCGAGGAGT
BRI1-GSO1-R1	ACTCCTCGCTTAGACCATTGGTAGCC
BRI1-BRL1-F1	TCTTGCTGCTTTCGAGAAACCGCTGAGAAA
BRI1-BRL1-R1	TCAGCGGTTTCTCGAAAGCAGCAAGATTGA
BRL1-R-BamHI	GGATCCAGGCTCCTTATCTCGCGATTCTTC
BRI1-BRL2-F1	TCTTGCTGCTTTCCAGCGACAGTTGAGAAA
BRI1-BRL2-R1	TCAACTGTCGCTGGAAAGCAGCAAGATTGA
BRL2-R-SalI	GTCGACCAAGCTGTTACTGTGACTGTGACT
BRI1-EMS1-F1	TCTTGCTGCTTTCGAGCAGCCGCTTC
BRI1-EMS1-R1	GAAGCGGCTGCTCGAAAGCAGCAAGA
EMS1-R-SalI	GTCGACTATCTCCTTAAGAGCCTTCAACAC
BRI1-$^{GSO1-S1}$-F1	ATATGAGTACATGAAGTATGGAAGTTTAG
BRI1-$^{GSO1-S1}$-R1	TCTTGTTTGACATACCGCTAACATGAATC
BRI1-$^{GSO1-S1}$-F	TCATGTTAGCGGTATGTCAAACAAGAG
BRI1-$^{GSO1-S1}$-R	AACTTCCATACTTCATGTACTCATATATC
BRI1-$^{GSO1-S2}$-F1	GATCGTGTTGATGGAGCTACTCACGGGTAA
BRI1-$^{GSO1-S2}$-R1	CTTCCATGTTTGAATCAAGCAACACATTAC

BRI1-$^{\text{GSO1-S2}}$-F	TGTGTTGCTTGATTCAAACATGGAAGCGCA
BRI1-$^{\text{GSO1-S2}}$-R	CCGTGAGTAGCTCCATCAACACGATCCCCA
GSO1-$^{\text{BRI1-S1}}$-F1	GTATGAGTTTATGAAGAACGGAAGCATCTG
GSO1-$^{\text{BRI1-S1}}$-R1	CTCTATCACCTTGGAGATCATCCTTCCAGA
GSO1-$^{\text{BRI1-S1}}$-F	GAAGGATGATCTCCAAGGTGATAGAGAGTT
GSO1-$^{\text{BRI1-S1}}$-R	TGCTTCCGTTCTTCATAAACTCATACACAA
GSO1-$^{\text{BRI1-S2}}$-F1	TGTGGTCTTACTCGAGATTGTGACTGGGAA
GSO1-$^{\text{BRI1-S2}}$-R1	CTTCCAAATTCTCATCGAGGAGCACATTAC
GSO1-$^{\text{BRI1-S2}}$-F	TGTGCTCCTCGATGAGAATTTGGAAGCTCG
GSO1-$^{\text{BRI1-S2}}$-R	CAGTCACAATCTCGAGTAAGACCACACCGT
BRI1-$^{\text{GSO1-C1}}$-F1	AGTAGGATTGGCTAGAGGGCTTGCTT
BRI1-$^{\text{GSO1-C1}}$-R1	CATGAAGCCAATCTTCTAAACTTCCA
BRI1-$^{\text{GSO1-C1}}$-F	TGGAAGTTTAGAAGATTGGCTTCATG
BRI1-$^{\text{GSO1-C1}}$-R	AAGCAAGCCCTCTAGCCAATCCTACT
BRI1-$^{\text{GSO1-C2}}$-F1	AAACATGGAAGCGCGGGTTTCAGATT
BRI1-$^{\text{GSO1-C2}}$-R1	AGTCATGATGAAGGAAAGCAAGCCCT
BRI1-$^{\text{GSO1-C2}}$-F	AGGGCTTGCTTTCCTTCATCATGACT
BRI1-$^{\text{GSO1-C2}}$-R	AATCTGAAACCCGCGCTTCCATGTTT
BRI1-$^{\text{GSO1-C3}}$-F1	TGCAGAGATGGACCTTGTTGGATGGG
BRI1-$^{\text{GSO1-C3}}$-R1	TCTCCATCAACACCACACCGTAACTA
BRI1-$^{\text{GSO1-C3}}$-F	TAGTTACGGTGTGGTGTTGATGGAGA
BRI1-$^{\text{GSO1-C3}}$-R	CCCATCCAACAAGGTCCATCTCTGCA
BRI1-$^{\text{GSO1-C4}}$-F1	TAAACTCATAGATCCCGAGCTTATGA
BRI1-$^{\text{GSO1-C4}}$-R1	CAACCCATCTCACAAGGTTGTTATCT

BRI1-$^{GSO1\text{-}C4}$-F	AGATAACAACCTTGTGAGATGGGTTG
BRI1-$^{GSO1\text{-}C4}$-R	TCATAAGCTCGGGATCTATGAGTTTA
BRI1-$^{GSO1\text{-}C5}$-F1	CCAAGAGAGACCAACAATGGTACAAG
BRI1-$^{GSO1\text{-}C5}$-R1	GAAGTGGCTTAAGCTCGGGGTCAAAC
BRI1-$^{GSO1\text{-}C5}$-F	GTTTGACCCCGAGCTTAAGCCACTTC
BRI1-$^{GSO1\text{-}C5}$-R	CTTGTACCATTGTTGGTCTCTCTTGG
GSO1$^{\text{-}BRI1\text{-}C1}$-F1	GATAGGATCAGCTCAAGGAGTAGAGT
GSO1$^{\text{-}BRI1\text{-}C1}$-R1	CGTGCAAAACATCCCAGATGCTTCCG
GSO1$^{\text{-}BRI1\text{-}C1}$-F	CGGAAGCATCTGGGATGTTTTGCACG
GSO1$^{\text{-}BRI1\text{-}C1}$-R	ACTCTACTCCTTGAGCTGATCCTATC
GSO1$^{\text{-}BRI1\text{-}C2}$-F1	GAATTTGGAAGCTCATTTAGGAGATT
GSO1$^{\text{-}BRI1\text{-}C2}$-R1	AGTTGTGGTGAAGGTACTCTACTCCT
GSO1$^{\text{-}BRI1\text{-}C2}$-F	AGGAGTAGAGTACCTTCACCACAACT
GSO1$^{\text{-}BRI1\text{-}C2}$-R	AATCTCCTAAATGAGCTTCCAAATTC
GSO1$^{\text{-}BRI1\text{-}C3}$-F1	TGGAGATAACAACATGGTGAGATGGG
GSO1$^{\text{-}BRI1\text{-}C3}$-R1	GCTCGAGTAAGACGATCCCCATACTG
GSO1$^{\text{-}BRI1\text{-}C3}$-F	CAGTATGGGGATCGTCTTACTCGAGC
GSO1$^{\text{-}BRI1\text{-}C3}$-R	CCCATCTCACCATGTTGTTATCTCCA
GSO1$^{\text{-}BRI1\text{-}C4}$-F1	CGATGTGTTTGACCCAAAGCTTAAGC
GSO1$^{\text{-}BRI1\text{-}C4}$-R1	TCACCCATCCAACCATGTCCATCTCT
GSO1$^{\text{-}BRI1\text{-}C4}$-F	AGAGATGGACATGGTTGGATGGGTGA
GSO1$^{\text{-}BRI1\text{-}C4}$-R	GCTTAAGCTTTGGGTCAAACACATCG
GSO1$^{\text{-}BRI1\text{-}C5}$-F1	TTGGAGACGACCGTCTTCTAGGCAAG
GSO1$^{\text{-}BRI1\text{-}C5}$-R1	CTTCCTTCATAAGCTTTGGATCTATG

GSO1$^{-BRI1-C5}$-F	CATAGATCCAAAGCTTATGAAGGAAG
GSO1$^{-BRI1-C5}$-R	CTTGCCTAGAAGACGGTCGTCTCCAA
GSO1$^{-BRI1-S1(N)}$-F1	ATCAAACATCGAAATCTGGTTAAGCTAATG
GSO1$^{-BRI1-S1(N)}$-R1	CATCTCCAAACCCTCCTGATCCAATC
GSO1$^{-BRI1-S1(N)}$-F	GATTGGATCAGGAGGGTTTGGAGATG
GSO1$^{-BRI1-S1(N)}$-R	CATTAGCTTAACCAGATTTCGATGTTTGAT
GSO1$^{-BRI1-S2(N)}$-F1	CCTTGTTGGATGGGTTGAAACACATC
GSO1$^{-BRI1-S2(N)}$-R1	TTTGGTAATACTCTGGAGCAATGTAG
GSO1$^{-BRI1-S2(N)}$-F	CTACATTGCTCCAGAGTATTACCAAA
GSO1$^{-BRI1-S2(N)}$-R	GATGTGTTTCAACCCATCCAACAAGG
GSO1$^{-BRI1-S2(S)}$-F1	CAACAAAAGGAGACGTTTACAGTATG
GSO1$^{-BRI1-S2(S)}$-R1	CTTTGGTAATACTCTGGAGCAATGTA
GSO1$^{-BRI1-S2(S)}$-F	TTGCTCCAGAGTATTACCAAAGT
GSO1$^{-BRI1-S2(S)}$-R	CTGTAAACGTCTCCTTTTGTTGA
BRI1-pGEX-F-BamHI	GGATCCGGTAGAGAGATGAGGAAGAGAC
BRI1-pGEX-R-SalI	GTCGACTCATAATTTTCCTTCAGGAACT
GSO1-pGEX-F-BamHI	GGATCCGTAATCGCTCTCTTCTTCAAACAA
GSO1-pGEX-R-SalI	GTCGACTTACAGCTTCTTATAACCGGCCGTTC
BRI1-KD^{E1078K}-F	GTGTGGTCTTACTCAAGCTACTCACGG
BRI1-KD^{E1078K}-R	CCGTGAGTAGCTTGAGTAAGACCACAC
Semi-quantitative PCR	
CPD-RT-F	GTTCTTATCCTGCTTCCATTTG

CPD-RT-R	AGCCACTCGTAGCGTCTCATT
DWF4-RT-F	CGAAGGAAGGCTCTTTGAATG
DWF4-RT-R	CTTCAACGGCTTTAGGGCAA
BAS1-RT-F	GTTCAGGACATTGTGGAGGAG
BAS1-RT-R	GGATAAAGCAACATAAGGACG
ACT2-RT-F	ACTCTCCCGCTATGTATGTCG
ACT2-RT-R	TGGACCTGCCTCATCATACTC
genotyping	
bri1-116-F	CCGGTTAACCGGTGAGATTCCGAAATGG
bri1-116-R	AGAATCTGCTTTGGCTCTGTTTCTAACT
